# Relevance of next generation sequencing (NGS) data re-analysis in the diagnosis of monogenic diseases leading to organ failure

**DOI:** 10.1186/s12920-023-01747-w

**Published:** 2023-11-27

**Authors:** Claudia Saglia, Valeria Bracciamà, Luca Trotta, Fiorenza Mioli, Angelo Corso Faini, Giulia Margherita Brach Del Prever, Silvia Kalantari, Maria Luca, Carmelo Maria Romeo, Caterina Scolari, Licia Peruzzi, Pier Luigi Calvo, Alessandro Mussa, Roberta Fenoglio, Dario Roccatello, Claudio Alberti, Diana Carli, Antonio Amoroso, Silvia Deaglio, Tiziana Vaisitti

**Affiliations:** 1 Immunogenetics and Transplant Biology Service, University Hospital “Città della Salute e della Scienza di Torino”, Turin, Italy; 2 Department of Medical Sciences, University of Turin, Turin, Italy; 3 GenomSys SA, Lausanne, Switzerland; 4 Pediatric Nephrology Dialysis and Transplantation Unit, University Hospital “Città della Salute e della Scienza di Torino”, Turin, Italy; 5 Pediatric Gastroenterology Unit, University Hospital “Città della Salute e della Scienza di Torino”, Turin, Italy; 6 Pediatric Clinical Genetics, University Hospital “Città della Salute e della Scienza di Torino”, Turin, Italy; 7 Department of Public Health and Pediatric Sciences, University of Turin, Turin, Italy; 8 Nephrology and Dialysis Unit, Center of Research on Immunopathology and Rare Diseases, CMID, San Giovanni Bosco Hospital, Turin, Italy; 9 Department of Clinical and Biological Sciences, University of Turin, Turin, Italy

**Keywords:** Clinical exome sequencing, Next-generation sequencing, Kidney Diseases, Liver Diseases, Genetic re-analysis, Variant re-classification, Re-alignment.

## Abstract

**Background:**

In 2018, our center started a program to offer genetic diagnosis to patients with kidney and liver monogenic rare conditions, potentially eligible for organ transplantation. We exploited a clinical exome sequencing approach, followed by analyses of in silico gene panels tailored to clinical suspicions, obtaining detection rates in line with what reported in literature. However, a percentage of patients remains without a definitive genetic diagnosis. This work aims to evaluate the utility of NGS data re-analysis for those patients with an inconclusive or negative genetic test at the time of first analysis considering that (i) the advance of alignment and variant calling processes progressively improve the detection rate, limiting false positives and false negatives; (ii) gene panels are periodically updated and (iii) variant annotation may change over time.

**Methods:**

114 patients, recruited between 2018 and 2020, with an inconclusive or negative NGS report at the time of first analysis, were included in the study. Re-alignment and variant calling of previously generated sequencing raw data were performed using the GenomSys Variant Analyzer software.

**Results:**

21 previously not reported potentially causative variants were identified in 20 patients. In most cases (*n* = 19), causal variants were retrieved out of the re-classification from likely benign to variants of unknown significance (VUS). In one case, the variant was included because of inclusion in the analysis of a newly disease-associated gene, not present in the original gene panel, and in another one due to the improved data alignment process. Whenever possible, variants were validated with Sanger sequencing and family segregation studies. As of now, 16 out of 20 patients have been analyzed and variants confirmed in 8 patients. Specifically, in two pediatric patients, causative variants were *de novo* mutations while in the others, the variant was present also in other affected relatives. In the remaining patients, variants were present also in non-affected parents, raising questions on their re-classification.

**Conclusions:**

Overall, these data indicate that periodic and systematic re-analysis of negative or inconclusive NGS data reports can lead to new variant identification or reclassification in a small but significant proportion of cases, with benefits for patients’ management.

**Supplementary Information:**

The online version contains supplementary material available at 10.1186/s12920-023-01747-w.

## Background

A significant proportion of end-stage organ failures, that in turn require organ transplantation, are caused by monogenic conditions, meaning that they are due to a single causative gene [1].

A major boost to the diagnosis of these monogenic diseases has been given by the technological advancement of next-generation sequencing (NGS)-based methods. Over the past decade, genomic technologies have dramatically evolved and NGS approaches are now widely applied to the diagnosis of inherited disorders of monogenic and polygenic nature [2, 3].

The diagnostic rate of NGS varies from 20 to 60% depending on several factors, including genetic and allelic heterogeneity of the disease, patients’ recruitment criteria, clinical presentation, sequencing platforms and laboratory analytical workflow [4, 5]. Despite technological advancements, a significant percentage of patients remains without a definitive molecular diagnosis. Both technical and clinical reasons may explain these results, including (i) failure of the analytical software to identify a variant due to a low allele frequency, poor sequencing and alignment quality, or filtering setting during the analytical process; (ii) absence of causative gene(s) within the in-silico disease panel adopted for the analysis because of lack of association with a given disease at the time of analysis; (iii) incorrect or incomplete clinical evaluation as in the presence of heterogenous phenotypes that may deviate from the classical phenotypes associated with certain diseases [6–8].

The aim of this work was to test the utility and clinical value of NGS data re-analysis, after at least two years from the first analysis, for those patients who received an inconclusive or negative genetic diagnosis. To this aim, a different analytical software was used for data alignment and variants calling compared to the first genetic analysis, together with variant segregation studies for an optimal variant(s) annotation. The rationale for the re-analysis rests on the continuous evolution of both data knowledge, including re-classification of existing causative variants, identification of new potentially causative variants, the steady pace of new disease gene associations, and diagnostic and analytical techniques [8–10]. Indeed, several studies show that an increase in diagnostic yield in NGS data re-analysis is linked to the discovery of new gene-disease and variant-disease associations, underlining the importance of periodic updating of gene lists adopted for the analysis based on public disease databases, such as OMIM, ClinVar and PanelApp [8, 11]. Furthermore, there is a constant upgrade of the available bioinformatics tools and variant prediction and classification databases. Variants previously annotated as likely benign or of unknown significance (VUS) can be re-classified and assigned to a class with a greater pathogenic impact. On the contrary, VUS or likely pathogenic variants may be downgraded based on new evidence. This temporal re-classification process is mainly the result of the sharing of genotypic-phenotypic data coming from different studies or Institutions, including large international consortia, finally allowing to shed light on the genetics of rare diseases and leading to the discovery of new putative disease-causing variants [12].

The discovery of novel genotype-phenotype associations through functional studies allows validation and redefinition of the clinical significance of VUS variants, thus, the time factor may play in favor of redefining the clinical relevance of variants of uncertain pathogenic significance [13–15].

At the light of this evidence, identifying a previously unrecognized genetic cause of monogenic disease is crucial (i) for patient’s management, (ii) to understand the risk of inheritance in patient’s family members, (iii) to recognize family members who carry the same genetic lesion, in case of transplantation from a living family donor [16]. Moreover, some forms of nephropathy and hepatopathy are at risk of recurrence after transplantation: having a genetic diagnosis can help to exclude the risk of recurrence [12, 17–19].

## Methods

### Patients’ recruitment

The study was based on a cohort of 114 patients referred to the Immunogenetics and Transplant Biology Service – Azienda Ospedaliero Universitaria Molinette Città della Salute e della Scienza, between October 2018 and March 2020, who resulted with a negative or inconclusive genetic analysis. Patients performed a clinical exome sequencing (CES) analysis based on the TruSight One expanded kit from Illumina (Illumina, San Diego, CA, United States) [19]. All patients included in the study provided an informed consent, that in the case of pediatric patients was signed by both parents, whenever possible. 106 patients have a suspected monogenic kidney disease, while the remaining 8 have a suspected monogenic liver disease. Detailed information on clinical suspicion, age at recruitment and family history is provided in Additional File 1 Table S1.

### Data alignment and analytical pipeline

NGS data from 114 patients, who tested negative or with an inconclusive report at the time of their first genetic analysis performed between October 2018 and March 2020, were re-aligned and re-analyzed using a new analytical software, the GenomSys Variant Analyzer (GVA; from GenomSys SA, Lausanne, Switzerland; https://genomsys.com/genomsys-variant-analyzer-experience/). This software allows both secondary and tertiary analyses. FASTQ files, generated by the Illumina sequencing platform NextSeq550, were used as input data, and genome sequencing read were aligned to a reference genome (hg19) using the bwa-mem2 aligner adapted to process and compress data to the MPEG-G format. Variant calling on aligned MPEG-G files were performed using the Freebayes software. Following tertiary analysis, for each sample, the VCF files were integrated with coverage tables, variant annotation table and a quality control report was produced that include parameters on input data quality, coverage and alignment [20]. The GVA software was validated by confirming the analyses with Illumina Variant Interpreter, the software routinely used for NGS analyses.

Variants annotation and curation was then performed in a step-by-step workflow based on the following criteria.

i) In-silico designed panels and subpanels comprising genes associated with diseases related to the patients’ clinical suspicion (Additional file 1 Tables S2-S3). These panels are updated and revised on an annual basis by querying genotype-phenotype databases, such as PanelApp England, PanelApp Australia, ClinGen, OMIM, Orphanet. The analytical pipeline included the possibility of extending the re-analysis to panels containing a larger number of genes, in case no variants were identified with subpanels.
ii) Sequencing and variants features. To limit the spectrum of variants identified to those with good sequencing quality and sufficient coverage, metrics filters were applied. Taking advantage of the software settings, variants that failed the quality filter (Filter Pass), whose allele frequency was lower than 0.1 and with an overall call depth lower than 10 reads were filtered out. Similarly, variants classified as benign (C1) or likely benign (C2) were also excluded, evaluating only likely pathogenic (C4) or pathogenic (C5) variants or those with an unknown significance (VUS-C3). Variant annotation by GVA was based on results displayed in Varsome, which is directly queried by GVA and sums up the verdicts of several meta-analyzers. Specifically, it comprises different databases that assessed variant frequency in the general population and in different ethnic groups (GnomAD), the degree of nucleotide or amino acid conservation (PhastCons, PhyloP and GERP), the impact of the variant on protein structure/function (SIFT, DANN, PolyPhen-2, MutationTaster, UniProt).

**Fig. 1:**
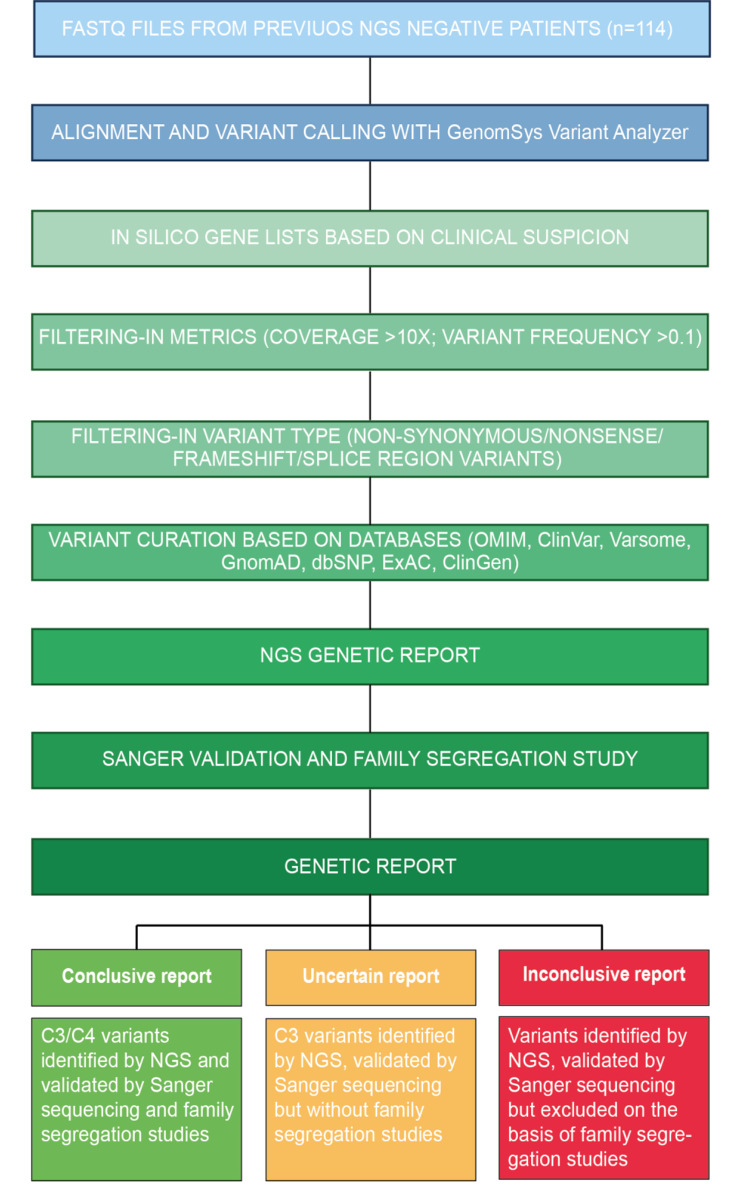
Workflow chart of the NGS data re-analysis and criteria for variants inclusion. Schematic representation of the analytical pipeline adopted for NGS data re-analysis of the 114 patients included in the study, with a negative genetic diagnosis at the time of the first analysis. Filtering criteria and databases used to classify variants are indicated. Identified variants were included in a NGS genetic report. Whenever possible, variant(s) validation and family segregation studies were performed for a better variant(s) annotation and reported in a genetic report. Genetic reports were considered as conclusive, uncertain, or inconclusive based on the indicated criteria. C3: variant of unknown significance; C4: likely pathogenic variant; C5: pathogenic variant; AR: autosomal recessive

Variants were classified based on the American College of Medical Genetics and Genomics (ACMG) criteria [9]. For the filtered-in variants, patients’ reads mapping quality was assessed through intra- and inter-run comparisons using the Interactive Genome Viewer (IGV) software (https://software.broadinstitute.org/software/igv/igv2.3). This step allowed us to exclude poor quality variants, potential technical sequencing artifacts or polymorphisms present in other patients.

iii) Variant curation according to genotype-phenotype features and presence in literature. Identified variants were then subjected to an additional filtering-in phase based on a manual curation inquiring different databases that assessed clinical annotation of the variants (ClinVar and LOVD3) [21, 22] and previous publications (PubMed and Mastermind) (Fig. 1).

New potentially causative variants, classified as C3, C4 and C5, were then subsequently validated by Sanger sequencing to rule out false positive or technical artifacts and, whenever possible, segregation studies were performed to define a mode of inheritance and to identify potential variant carriers and *de novo* acquired mutations. This analysis was useful in verifying whether the identified variant segregated with the disease, being present in additional affected family members and absent in healthy subjects (Fig. 1). Moreover, the results of these analyses were used for an additional round of variants curation before including them in the final genetic report.

In cases where the NGS analysis concludes with a negative or inconclusive outcome, the FASTQ and VCF data were archived and after approximately two years, will be re-analyzed as re-classification of variants, the appearance of new scientific evidence and/or functional studies reported in the literature and the updating of population databases may occur after some time [23].

### Sanger sequencing

Primers were designed on the reference genome (hg19) using the Pick primers tool (https://www.ncbi.nlm.nih.gov/tools/primer-blast) and are listed in Additional file 1 Table S4. For *PKD1* variants validation, a long-PCR protocol for the amplification of the *PKD1* gene to exclude the 6 highly homologous pseudogenes was exploited starting from 60 ng of DNA template, and a reaction mix consisting of water, Buffer 5x, dNTPs, external primer pair, high processivity Taq Polymerase in the presence of Betaine and DMSO to access GC-rich regions (Expand™ Long Template PCR System, Roche, Milan, Italy). Primers used in the LR-PCR reactions were designed in the non-homology regions of the *PKD1* gene [24]. Amplicons were run in gels and a nested PCR reaction performed on the purified long-PCR products, using internal primers specific for the exon of interest [24].

Sanger sequencing reaction was then performed using the Big Dye Terminator v1.1 kit according to Manufacturer’s instructions (BigDye Terminator v1.1 Cycle Sequencing Kit, ThermoFisher Scientific, Milan, Italy). Sequencer generated binary files (ABIF - Applied Biosystems Inc. Format files) were subsequently analyzed using the open-access tool Chromas Plite 2.6 (https://chromas.software.informer.com/2.6/). In case of a discrepancy between Sanger sequencing and NGS results, DNA was purified again from an independent blood aliquot and Sanger sequencing repeated.

Whenever possible, segregation studies on proband and parents were performed to better understand the mode of inheritance of identified variants and classify their risk and pathogenic impact.

## Results

### Main characteristics of study cohorts

Two study cohorts (kidney and liver) were included in the analyses, comprising patients referred to the Immunogenetics and Transplant Biology Service of the Molinette Hospital in Turin (Azienda Ospedaliera Universitaria Città della Salute e della Scienza) who received a negative or inconclusive genetic report at the time of first analysis, performed between October 2018 and March 2020. Overall, in this timeframe, a total of 226 patients were analyzed by CES, of whom 208 (92%) diagnosed with a kidney disease and 18 (8%) with hepatopathies. In line with patients’ distribution and considering a diagnostic rate of approximately 50–60% [19], the re-analysis was performed on 106 patients referred for kidney diseases and 8 for liver diseases.

**Fig. 2:**
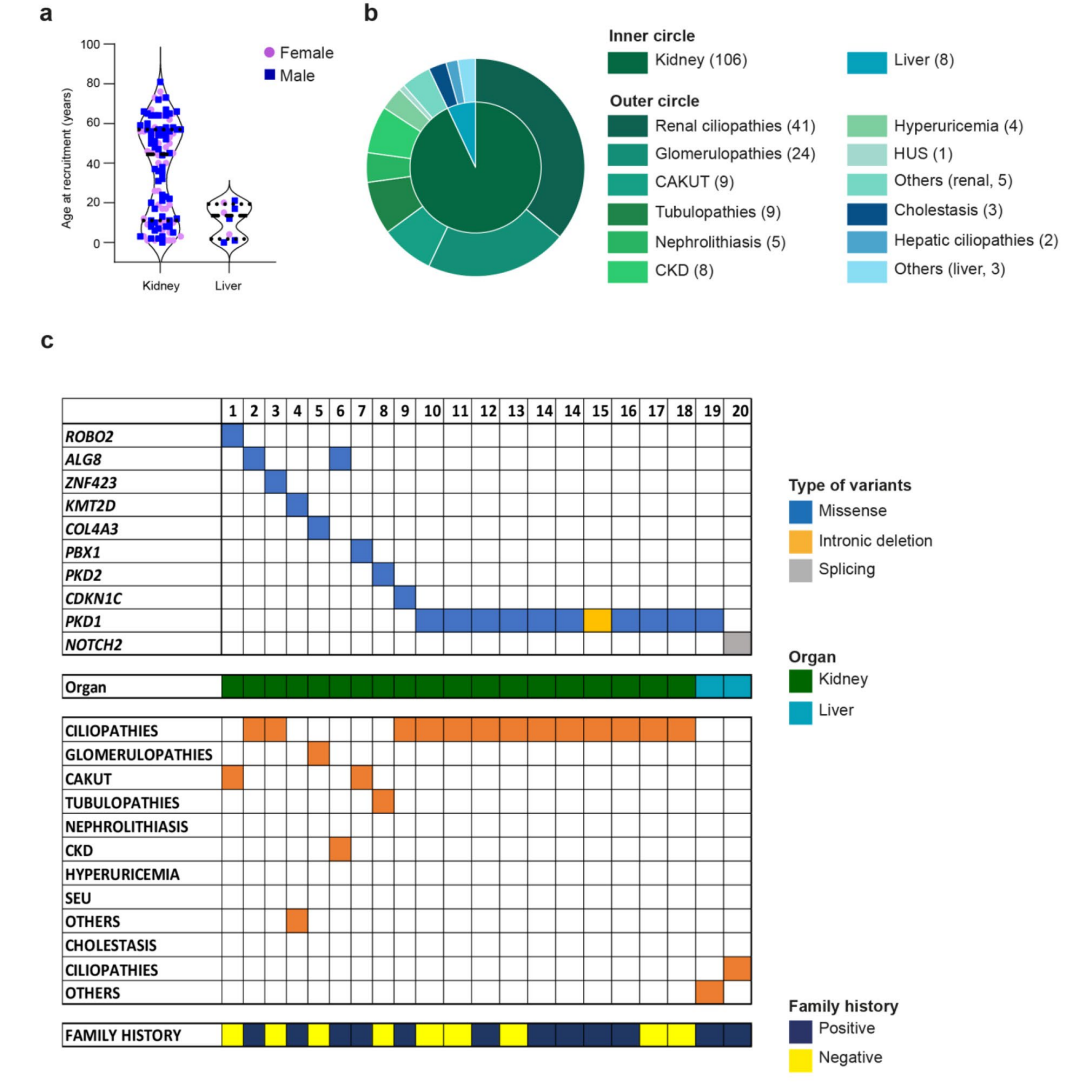
Main features of the study cohort and variants identified by NGS data re-analysis. Patients’ distribution according to their gender, age at recruitment and organ involved (kidney or liver). Data are shown as a violin plot: purple dots represent female subjects while blue square represent male patients (**a**). Patients’ distribution based on the main disease macro-categories (outer circle), considering renal and hepatic pathologies (inner circle, **b**). Summary of curated variants in disease-causative genes identified by NGS data re-analysis. Type of variant, organ, disease macro-category and family history are reported (raw) for each patient (column; **c**)

The kidney cohort included 58 males (55%) and 48 females (44%) with an average age of about 35 years and a predominance of pediatric (0–18 years old; *n* = 36; 34%) and over 50 years (*n* = 33; 31%; Fig. 2a) patients.

Based on the clinical suspicion, patients were divided into different macro-categories, including renal ciliopathies which represented the largest proportion of cases (*n* = 41; 36%), followed by glomerulopathies (*n* = 24; 21%), congenital anomalies of the kidney and urinary tract (CAKUT) and tubulopathies (*n* = 9; 8% each category), chronic kidney disease (CKD; *n* = 8; 7%), nephrolithiasis (*n* = 5; 4%), hyperuricemia (*n* = 4; 3%) and hemolytic uremic syndrome (HUS; *n* = 1; 0.8%). The remaining cases (*n* = 5; 4%), indicated as other, presented with syndromic features with predominantly renal symptomatology and several comorbidities.

Also in the liver cohort, male subjects were prevalent (*n* = 5; 62%) compared to females (*n* = 3; 38%). The average age was about 11 years, and patients can be placed in only two age groups: the most represented was the pediatric age group (*n* = 6; 75%) followed by the age group 18–35 years (*n* = 2; 25%). The cohort was divided into macro-categories with a clinical suspicion of cholestasis (*n* = 3; 30%), hepatic ciliopathy (*n* = 2; 20%), and others, that included hepatopathies of unspecified etiology and/or unclear syndromic pattern (*n* = 3; 30%) (Fig. 2b).

### Identification of genetic variants by NGS data re-analysis

Re-analysis of NGS data from 114 patients identified 21 new potentially causative variants in 20 different cases with a detection rate of 17.5% (Table 1; Fig. 2c).

The majority of the newly identified variants were missense (*n* = 19; 91%), one was an intronic deletion and one variant affected a splicing acceptor site (Fig. 2c). 80% (*n* = 17) of the new NGS variants were classified as VUS based on the ACMG classification criteria. Since most of them were missense variants, it was difficult to predict their impact at the protein level only based on bioinformatics tools. It was therefore essential, whenever possible, to subsequently perform family segregation studies to (re)define the pathogenetic significance of these variants.

The highest percentage of variants was identified in patients having a clinical suspicion of renal ciliopathies with 13 new potentially causative variants in 12 patients (Fig. 2c). Most of them (10 variants) were in *PKD1*, the main responsible gene for polycystic kidney. Out of these 12 patients, seven (58%) had a positive family history of polycystic kidney disease, underlining the relevance of re-analyzing patients who have a positive family history and resulted negative at the first analysis.

**Table 1:**
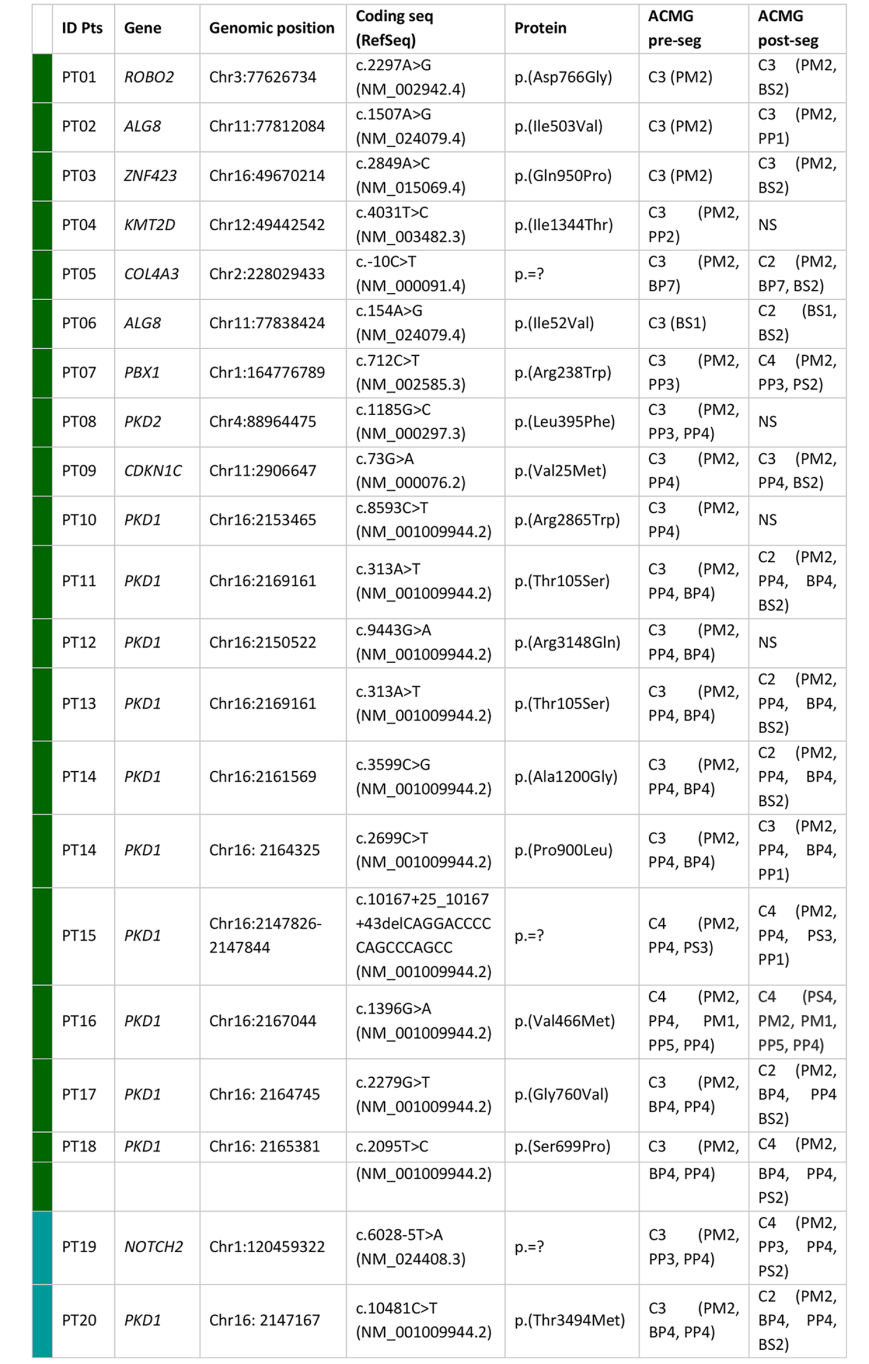
Variants identified by NGS data re-analysis. List of variants identified re-analyzing NGS data using the GenomSys software. Raw sequencing data were re-aligned and variants calling performed. Green square refers to patients affected by kidney diseases; light blue square refers to patients affected by liver diseases. All variants were identified in heterozygous state.ID Pts: patient identification code; Coding seq: coding sequence; RefSeq: reference sequence; ACMG: American College of Medical Genetics and Genomics; pre-seg: pre-segregation; post-seg: post-segregation; del: deletion; C4: Likely pathogenic variant; C3: variant of unknown significance (VUS); C2: likely benign; NS: not segregated

**Fig. 3:**
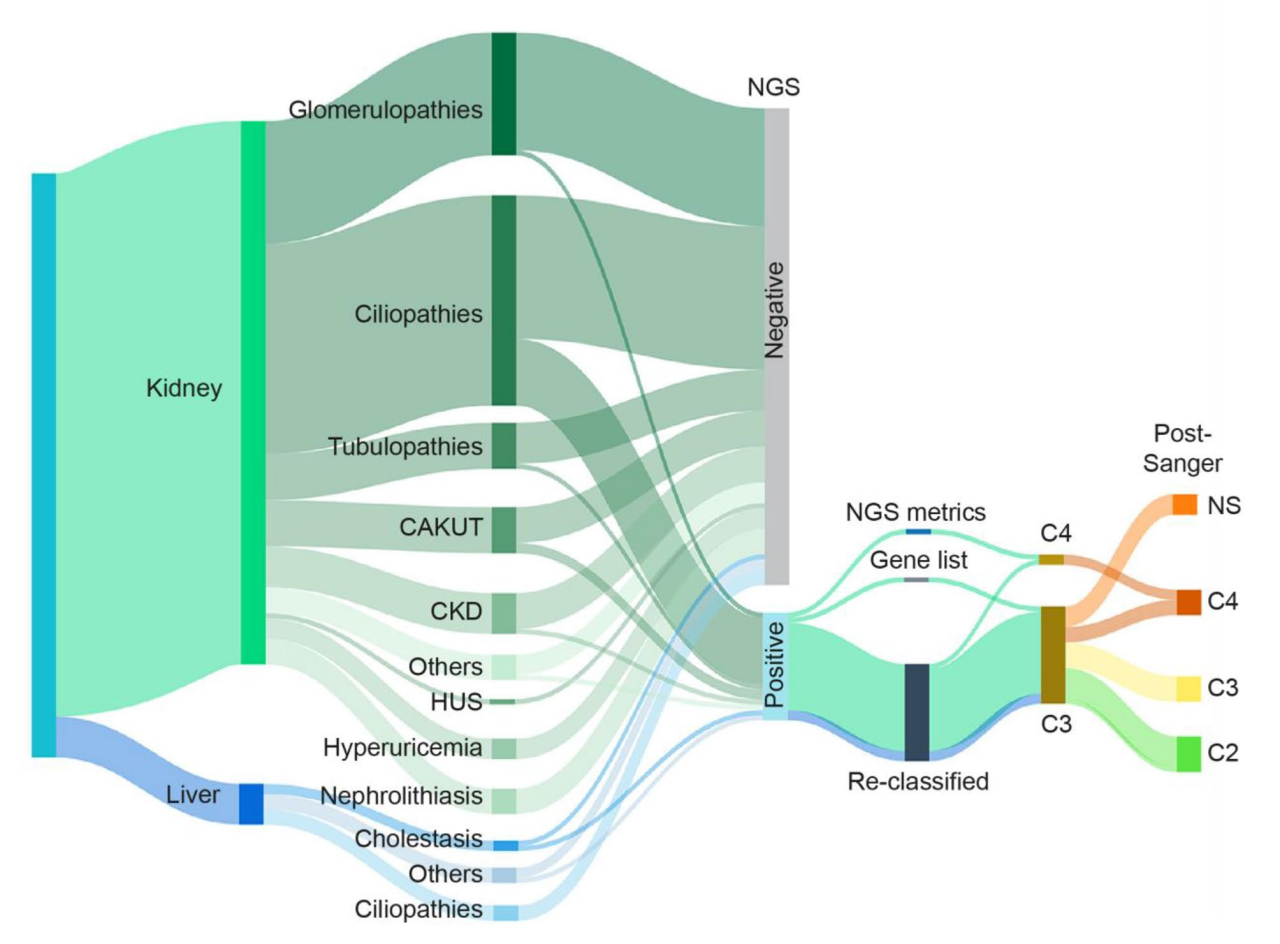
Schematic representation of the workflow leading to the identification of previously unidentified causative variants. Sankey diagram summarizing the distribution of the cohort and the results of the analytical workflow. Nodes represent: (i) organ of interest (kidney or liver), (ii) main disease macro-categories, (iii) variant(s) identification by NGS, (iv) reasons why variant was identified by re-analysis (metrics adopted during the re-analysis, gene lists update, variants re-classification), (v) variant classification post-NGS and (vi) variant classification post-Sanger and family segregation studies. Arrows thickness is proportional to the number of patients. The Sankey Matic tool was used to obtain the Sankey diagram. CAKUT: congenital abnormalities of kidney and urinary tract; CKD: chronic kidney disease; HUS: hemolytic uremic syndrome; NS: not segregated

We then delved into the reasons why these variants were not reported at the time of the first NGS analysis. For most of them, a re-classification to VUS or likely pathogenic variants occurred over time. Indeed, 90% of variants identified during the re-analysis were previously classified as likely benign and thus filtered out. In one case, a VUS variant was identified in the *PBX1* gene, a gene not originally included in the in-silico gene list investigated (CAKUT) and only recently recognized as causative for CAKUT disorders [25]. This finding underlines the utility of a yearly-based update of disease-associated gene lists through a systematic review of the literature and genotype-phenotype databases such as PanelApp, OMIM, Malacards. Finally, in one case, re-analysis identified a likely pathogenic intronic variant in *PKD1* due to data re-alignment and filtering metrics adopted during the tertiary analysis. The variant was indeed filtered-out in the previous analysis as it maps slightly out of the target regions (Fig. 3).

### Validation of identified variants and presentation of eight “genetically solved” patients

Validation of the newly identified variants and family segregation analyses were performed by Sanger sequencing, whenever possible. This latter type of analysis is critical to correctly classify the variants and clarify their role as causative mutations, specifically when VUS are identified and there is no evidence in literature.

This approach allowed to narrow the number of variants with a potential causative role as those identified in patients PT05, PT06, PT11, PT13, PT17, PT20 in *COL4A3, ALG8*, and *PKD1* genes, respectively, were downgraded from VUS to likely benign variants, as they were present and inherited from family members in apparent good health, and therefore labelled as not relevant to the phenotype (Figs. 2c and 3). The variants in *ROBO2*, *ZNF423*, and *CDKN1C* identified in patients PT01, PT03 and PT09, although present in a healthy parent, are still currently classified as VUS (Table 1). On the contrary, family segregation studies allowed to confirm the involvement of identified variants in causing the clinical phenotype in 5 patients, PT07, PT15, PT16, PT18, PT19 (*PBX1, PKD1* and *NOTCH2* genes, respectively) and to re-classify them as likely pathogenic variants (Fig. 3; Table 1). Variants identified in patients PT02 and PT14 (*ALG8* and *PKD1*) although present in an affected parent are still classified as VUS (Table 1). Finally, in 4 patients, variant classification remained of uncertain significance and thus reported as VUS because family segregation studies were unfeasible (PT04, PT08, PT10, PT12). In patients where a *PKD1* variant was found, validation by Sanger sequencing was extremely important. Indeed, this gene, located in a duplicated segmental region, presents with several pseudogenes that share 97.6–97.8% sequence homology with the gene. This duplicated region represents a diagnostic challenge for ADPKD, as conventional sequencing may be not effective in specifically targeting *PKD1* regions. Thus, verifying the presence of identified variants within the gene represent a critical step in the diagnostic workflow. In all patients of this study cohort in which a *PKD1* variant was identified, Sanger sequencing confirmed the presence of the variant.

Given the phenotypic relevance of the identified, confirmed, and reclassified variants and their importance for the genetic and clinical communities, we will present and discuss below in more detail the clinical presentation and symptomatology of these patients and how family studies were diriment for variant classification.

PT02: 32-year-old male patient presenting with renal cysts and liver microcysts. Re-analysis identified a variant of uncertain significance in *ALG8* (#608,103), a gene associated with kidney and liver cysts with an autosomal dominant pattern (https://omim.org/entry/617874), potentially compatible with patient’s clinical phenotype. Family segregation of the variant [chr11:77812084-T-C; c.1507 A > G; p.(Ile503Val)] in the *ALG8* gene revealed the presence of the same variant in his daughter, who also suffers from renal cysts but, at least till the time of analysis, without liver cysts. It has to be noted that the daughter is still in a pediatric age and therefore it may be too early for liver involvement. These results support the hypothesis that this variant, reported by the analytical software and Varsome as VUS, may be potentially causative of the clinical phenotype of the patient and his daughter. However, we have not achieved the degree of certainty necessary to reclassify it as a likely pathogenic variant.

**Fig. 4:**
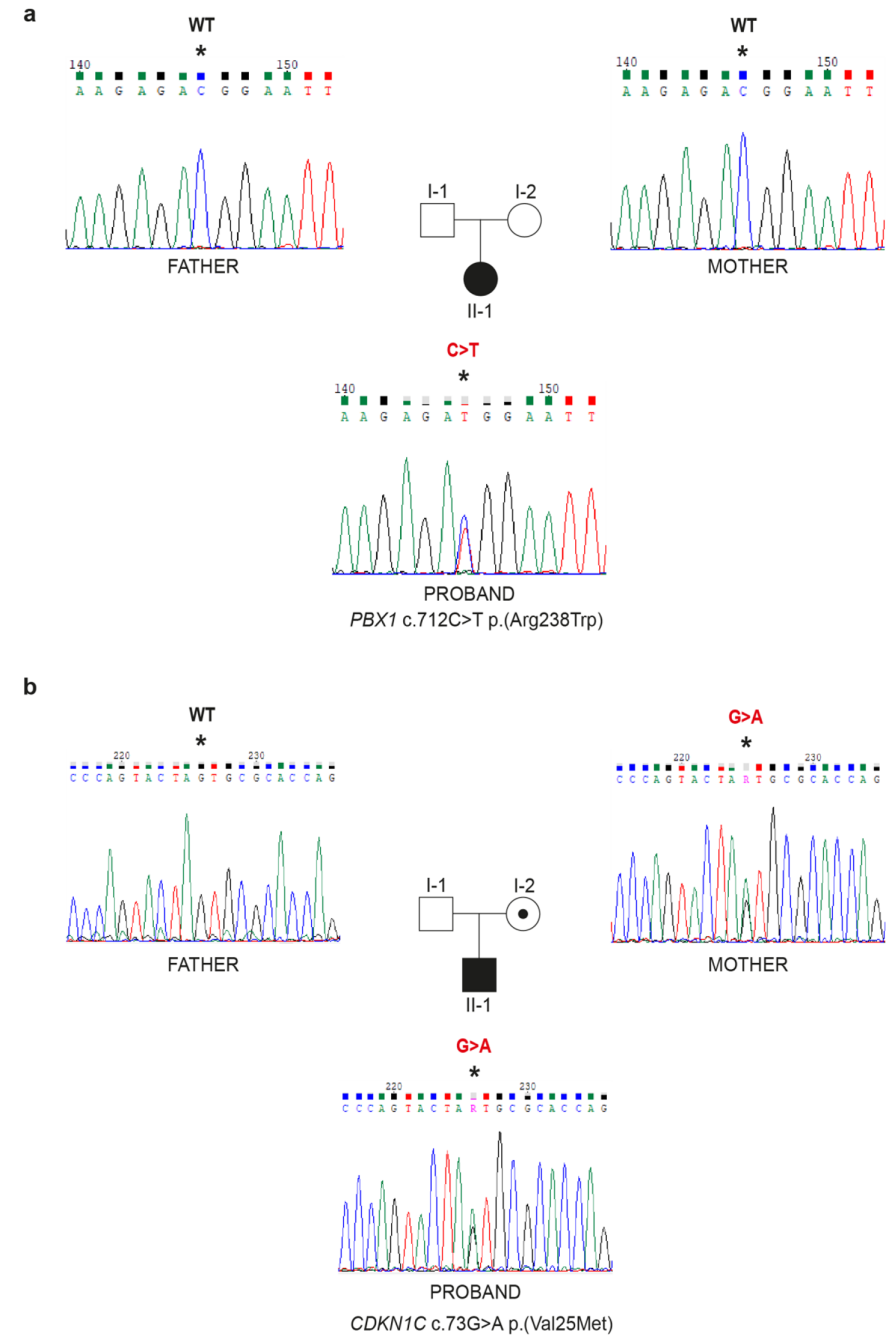
Examples of genetic diagnosis obtained by NGS data re-analysis. Chromatograms and family tree of 2 patients where a causative variant was identified by NGS data re-analysis and validated by Sanger sequencing and family segregation study. Proband had a *de novo* heterozygous variant in *PBX1*, as shown by a double peak in the Sanger electropherogram of the proband, while both parents were wild-type (**a**). Proband had a heterozygous missense variant in *CDKN1C*, as shown by a double peak in the Sanger electropherogram, which was inherited from the mother (**b**). Asterisk indicates the nucleotide affected. WT: wild-type

PT07: Syndromic pediatric patient presenting with CAKUT, minor growth and cognitive retardation and hypotonia, in the absence of a positive family history. Re-analysis of a panel of genes associated with CAKUT revealed a missense variant in *PBX1* (#617,641), which is associated with a complex syndromic pattern characterized by abnormalities of the kidney and urinary tract, cognitive retardation (https://omim.org/entry/617641) with an autosomal dominant mode of inheritance. These clinical features are compatible with the patient’s phenotype. The identified variant [chr1:164776789-C-T; c.712 C > T; p.(Arg238Trp)] in *PBX1* gene is reported as C3 in Varsome, while being absent in ClinVar. Sanger sequencing and segregation analysis confirmed the presence of the variant in the proband and excluded its presence in her parents indicating that this is a *de novo* variant, exclusively present in the affected proband. This variant was classified as VUS by both analysis software and Varsome because, although most predictors return an unfavorable score, no publications and functional evidence are reported. The segregation analysis performed in this study corroborates the clinical role of this variant and allows for a reclassification from a variant of uncertain significance to a likely pathogenic one (Fig. 4a; Table 1).

PT09: 2-year-old male referred for genetic analysis with a clinical suspicion of Beckwith-Wiedemann syndrome with mild macroglossia and frontal angioma at birth and kidney cysts. Genetic analysis performed looking at ciliopathies-associated genes was negative, but when the analysis was extended to the entire panel of genes associated with monogenic renal diseases, a variant in *CDKN1C* gene (#600,856) was found, a gene associated with Beckwith-Wiedemann syndrome with autosomal dominant transmission (https://omim.org/entry/130650). This missense variant [chr11:2906647-C-T; c.73G > A; p.(Val25Met)] in the *CDKN1C* is classified as VUS both in Varsome and ClinVar. Familial segregation study revealed the presence of the variant in patient’s mother and maternal grandmother (Fig. 4b). The mother was in apparent healthy conditions without any signs of the syndrome, thus inconsistent with the mode of inheritance of the disease. Indeed, *CDKN1C*, which codes for p57 (KIP2) protein, a potent inhibitor of several cyclin complexes and negative regulator of cell proliferation, is paternally imprinted with preferential expression of the maternal allele [26, 27].

PT14: 10-year-old female suffering from polycystic kidney disease (https://www.omim.org/entry/601313) with multiple bilateral cysts with a positive maternal family history. The mother manifested the symptoms in adulthood, while the daughter (the proband) showed an early onset at the age of nine years old. Re-analysis of CES data revealed two missense variants, [chr16:2164325-G-A; c.2699 C > T; p.(Pro900Leu) and chr16:2161569-G-C; c.3599 C > G; p.(Ala1200Gly)] both of uncertain significance, in the *PKD1* gene (#601,313). Family segregation study showed a maternal inheritance of the c.2699 C > T p.(Pro900Leu) variant, consistent with the family history reported. In contrast, the c.3599 C > G p.(Ala1200Gly) variant appeared to be inherited from the father, who is in apparent good health conditions. The analysis of the family pedigree allowed us to show that these two variants, identified in the proband, map on different alleles, with the maternally inherited one being possibly associated with patient clinical phenotype and but not reaching enough evidence to be reclassified as likely pathogenic. We then speculated that the second variant, inherited from the father, can be a hypomorphic variant that, together with the presence of the c.2699 C > T p.(Pro900Leu) variant, may exacerbate the phenotype, potentially justifying the earlier onset of the disease in the proband.

PT15: 57-year-old male with multiple kidney cysts, compatible with polycystic kidney disease, showing a positive family history since her father presented the same clinical phenotype, and died because of the disease. Re-analysis identified a 19-nucleotide intronic deletion within intron 31 (chr16:2147826–2,147,844; c.10,167 + 25_10167 + 43delCAGGACCCCCAGCCCAGCC) in *PKD1*, reported as likely pathogenic in Varsome and ClinVar. This variant was not reported in the previous analysis because of analytical reasons. Sanger validation and family segregation study of the variant confirmed the presence within the proband and showed its presence also in her daughter, who suffers from multiple renal cysts, as shown by ultrasonographic sound test. These data confirmed the pathogenicity of the variant which is causative of the clinical phenotype of this family.

PT16: 61-year-old patient with chronic kidney disease stage II, as a consequence of a suspected polycystic disease, presenting a positive family history of renal cystic disease (maternal lineage). For this patient, re-analysis revealed a *PKD1* genetic variant [chr16:2165381-A-G; c.1396G > A; p.(Val466Met)], previously excluded from the report as it was classified as likely benign and thus filtered-out. Validation by Sanger sequencing and familial segregation in the proband’s daughters (two) showed that the variant is present in the daughter presenting with cystic kidney disease, whereas it is absent in the healthy one. These results allowed us to confirm the pathogenetic role of the variant and to classify it as likely pathogenic.

PT18: 56-year-old female patient affected by polycystic kidney disease, with no family history. In this case, re-analysis revealed a VUS variant [chr16:2165381-A-G; c.2095T > C; p.(Ser699Pro)] in *PKD1*, not previously reported as classified as likely benign, and Sanger validation and family segregation study allowed to confirm its presence exclusively in the proband, as no variants were identified in her parents and sister, who are all in apparent good health conditions. These data strongly support a re-classification of the variant from VUS to likely pathogenic.

PT19: 4-year-old female patient diagnosed with a syndromic disease compatible with Alagille syndrome, characterized by cognitive retardation, dysmorphic facies, heart defects, rib anomalies, and hepatopathy. Re-analysis of CES data with an in-silico gene list consisting of the entire panel of genes associated with monogenic liver disease (289 genes), allowed for the identification of a splicing variant [chr1:120459322-A-T; c.6028-5T > A] in *NOTCH2* gene (#600,275), which is associated with Alagille syndrome (https://omim.org/entry/610205). The variant is reported as likely pathogenic in Varsome while absent in ClinVar. The c.6028-5T > A variant is located within the splicing region of intron 33 and is predicted to impact the splicing process, being responsible for the clinical phenotype of the patient. Family segregation ascertained the absence of the variant in proband’s parents, confirming its *de novo* nature.

## Discussion

Although clinical exome sequencing has become a powerful tool in the diagnostic workflow of monogenic diseases both in children and adults, detecting disease-associated variants is a complex process that sometimes turns out to be unsuccessful [19, 28–30]. Indeed, depending on several parameters of the sequencing and analytical process as well as on criteria adopted for patients recruitment, 40–60% of the samples remain unsolved at the time of the first genetic analysis [31, 32]. These data pose different questions and open to the need of re-analyzing NGS data after a period of time, which has to be defined on an empirical and clinical base [33–35]. In the last years, evidence has come out showing the relevance of data re-analysis, but no guidelines from the relevant scientific societies have been yet published [36–39].

The results obtained in this work showed that re-analysis of the NGS data of a cohort of 114 patients, both children and adults, identified 21 new variants compatible with the clinical phenotype in 20 different patients, with a total detection rate of 17.5%. Comparing these data with those of the first analysis, performed between October 2018 and March 2020, a 7% increase in the diagnostic rate was obtained with the second analysis. The discovery of new disease-associated genes together with the re-classification of variants and extended phenotypic information that led to less stringent filtering strategies, contributed to this increased diagnostic rate, corroborating the need for a periodic re-analysis of NGS data. Most of the variants identified by the re-analysis were highlighted because of reclassification from likely benign (C2) to VUS (C3) and only in three conditions from C2 to a more deleterious pathogenic class (C4; PT07, PT16 and PT18). It must be noted also that the use of novel and improved tools for NGS data analysis, specifically for data re-alignment, may be critical in obtaining a better mapping of the reads thus highlighting variants not previously detected.

A critical point in the re-analysis process was Sanger validation together with family segregation studies that helped to confirm or redefine the pathogenic significance of the identified variants. These combined approaches allowed us to exclude from the final genetic report the variants identified in 8 patients, as these variants were also detected in non-affected subjects. On the contrary, in 4 patients, variant classification was redefined from VUS to likely pathogenic as they were uniquely found in affected family members, and thus, they were causative of the disease. Only for four patients, PT04, PT08, PT10, and PT12, the family study could not be carried out to confirm the pathogenicity of variants. Furthermore, the family segregation study established that two variants in two pediatric patients, PT07 and PT19, were *de novo* acquired. This information was critical for the family and to calculate the risk of inheritance in case of another pregnancy, although germline mosaicism could not be ruled out.

The results obtained by the NGS data re-analysis are important not only for research purposes, but also for patients and their families, as a conclusive genetic diagnosis constitutes a first and fundamental step in the diagnostic and therapeutic process. Being aware of the causative gene of a disease and the potential impact of the variant on the protein makes it possible to predict a prognostic course and establish more appropriate screening and prevention measures. In addition, genetic diagnosis is critical to puzzle-out complex or heterogeneous phenotypes, as in the case of patient PT14 where two variants in *PKD1* were highlighted following re-analysis, potentially determining the early onset of the disease and, for a correct patient’s management [40]. Thus, the resolution of a non-diagnostic case brings with it an improvement in both physical and psychological aspects for the patient.

A final consideration regards the time- and cost-impact of NGS data re-analysis. As in our case, NGS for the diagnosis of monogenic diseases is part of a routine public diagnostic service with a continuous patient enrollment. Performing a periodic re-analysis and making it part of the diagnostic workflow represents a significant human and economic effort and thus, the feasibility may depend on the available local infrastructure, bioinformatics support, specialized dedicated personnel, and budget.

## Conclusions

Although a considerable number of cases remains negative or inconclusive even at re-analysis, the 7% increase in the diagnostic rate obtained in this work supports the value of a periodic re-analysis of sequencing data as a powerful tool for detecting new variants or variants in new disease-associated genes. Moreover, we highlighted the importance of combined segregation studies to better define the pathogenic significance of identified variants. A still open point, not yet addressed by the relevant scientific societies, is the timeframe to perform a re-analysis from the preceding one and how this process can fit with the diagnostic routine of NGS services operating within the national health system.

Periodic re-analysis of the NGS data, together with segregation familial segregation, could change the clinical course of the disease or patient’s management and put an end to what often becomes a diagnostic odyssey for the patient and family in the case of undiagnosed disease.

### Electronic supplementary material

Below is the link to the electronic supplementary material.

Supplementary Material 1

## Data Availability

The datasets generated and/or analyzed during the current study are available in the ClinVar repository [SCV004040457-SCV004040467-SCV004037594-SCV004037601].

## Abbreviations

NGS: Next-generation sequencing
CKD: Chronic kidney disease
ESRD: End-stage renal disease
IKD: Inherited kidney disease
ACMG: American College of Medical Genetics and Genomics
CES: Clinical exome sequencing
VUS: Variants of unknown significance
CAKUT: Congenital abnormalities of kidney and urinary tract
HUS: Hemolytic uremic syndrome
