# Supplementary material for: Relevance of next generation sequencing (NGS) data re-analysis in the diagnosis of monogenic diseases leading to organ failure
Source: BMC Med Genomics. 2023 Nov 27;16:303. doi: 10.1186/s12920-023-01747-w (PMC10680258; doi:10.1186/s12920-023-01747-w)
Supplement: Supplementary file 1 — Supplementary Material 1 [file 12920_2023_1747_MOESM1_ESM.docx]

**Additional file 1**

|  |  |  |  |  |  |  |  |  |
| --- | --- | --- | --- | --- | --- | --- | --- | --- |
|  | **Table S1. Main features of the study cohort.** M: male; F: female; CKD: chronic kidney disease; HUS: Hemolytic-Uremic Syndrome | | | | | | | |
|  | **Patient ID** | **Gender** | **Age at recruitment (y.o.)** | **Organ** | **Clinical suspicion** | **Disease macro-category** | **Family history** | **Re-analysis** |
|  | PT01 | M | 17 | Kidney | CAKUT | CAKUT | No | *ROBO2* |
|  | PT02 | M | 32 | Kidney | Renal failure | CKD | Yes | *ALG8* |
|  | PT03 | F | 1 | Kidney | Polycystic kidney | Ciliopathies | No | *ZNF423* |
|  | PT04 | M | 3 | Kidney | Syndromic disease | Other | Yes | *KMT2D* |
|  | PT05 | M | 12 | Kidney | Focal segmental glomerulosclerosis | Glomerulopathies | No | *COL4A3* |
|  | PT06 | M | 36 | Kidney | Polycystic kidney | Ciliopathies | Yes | *ALG8* |
|  | PT07 | F | 3 | Kidney | Syndromic disease | CAKUT | Yes | *PBX1* |
|  | PT08 | M | 35 | Kidney | Tubulopathy | Tubulopathies | No | *PKD2* |
|  | PT09 | M | 2 | Kidney | Cystic kidney disease | Ciliopathies | Yes | *CDKN1C* |
|  | PT10 | M | 0 | Kidney | Cystic kidney disease | Ciliopathies | No | *PKD1* |
|  | PT11 | M | 66 | Kidney | Cystic kidney disease | Ciliopathies | No | *PKD1* |
|  | PT12 | F | 6 | Kidney | Polycystic kidney | Ciliopathies | No | *PKD1* |
|  | PT13 | F | 1 | Kidney | Cystic kidney disease | Ciliopathies | No | *PKD1* |
|  | PT14 | F | 10 | Kidney | Polycystic kidney | Ciliopathies | Yes | *PKD1* |
|  | PT15 | M | 57 | Kidney | Polycystic kidney | Ciliopathies | Yes | *PKD1* |
|  | PT16 | M | 57 | Kidney | Polycystic kidney | Ciliopathies | No | *PKD1* |
|  | PT17 | F | 58 | Kidney | Cystic kidney disease | Ciliopathies | Yes | *PKD1* |
|  | PT18 | F | 56 | Kidney | Polycystic kidney | Ciliopathies | Yes | *PKD1* |
|  | PT19 | M | 12 | Liver | Syndromic disease | Other | Yes | *NOTCH2* |
|  | PT20 | F | 4 | Liver | Cystic kidney disease | Ciliopathies | Yes | *PKD1* |
|  | PT21 | M | 65 | Kidney | Glomerulonephritis | Glomerulopathies | Yes | Negative |
|  | PT22 | M | 60 | Kidney | Renal failure | CKD | No | Negative |
|  | PT23 | F | 62 | Kidney | Fabry disease | Other | Yes | Negative |
|  | PT24 | M | 44 | Kidney | Focal segmental glomerulosclerosis | Glomerulopathies | Yes | Negative |
|  | PT25 | M | 64 | Kidney | Focal segmental glomerulosclerosis | Glomerulopathies | Yes | Negative |
|  | PT26 | F | 40 | Kidney | Renal failure | CKD | Yes | Negative |
|  | PT27 | M | 50 | Kidney | Nephrotic syndrome | Glomerulopathies | Yes | Negative |
|  | PT28 | M | 22 | Kidney | Gitelman syndrome | Tubulopathies | No | Negative |
|  | PT29 | M | 58 | Kidney | Hyperuricemia | Hyperuricaemia | Yes | Negative |
|  | PT30 | M | 37 | Kidney | Focal segmental glomerulosclerosis | Glomerulopathies | Yes | Negative |
|  | PT31 | F | 19 | Kidney | Focal segmental glomerulosclerosis | Glomerulopathies | No | Negative |
|  | PT32 | F | 50 | Kidney | Gitelman syndrome | Tubulopathies | No | Negative |
|  | PT33 | M | 54 | Kidney | C3 glomerulopathy | Glomerulopathies | No | Negative |
|  | PT34 | M | 13 | Kidney | Focal segmental glomerulosclerosis | Glomerulopathies | No | Negative |
|  | PT35 | F | 17 | Kidney | Focal segmental glomerulosclerosis | Glomerulopathies | No | Negative |
|  | PT36 | F | 19 | Kidney | C3 glomerulopathy | Glomerulopathies | No | Negative |
|  | PT37 | M | 59 | Kidney | Focal segmental glomerulosclerosis | Glomerulopathies | Yes | Negative |
|  | PT38 | F | 1 | Kidney | Nephrolithiasis | Nephrolithiasis | No | Negative |
|  | PT39 | F | 64 | Kidney | Bartter's syndrome | Tubulopathies | No | Negative |
|  | PT40 | F | 55 | Kidney | Renal failure | CKD | No | Negative |
|  | PT41 | F | 3 | Kidney | CAKUT | CAKUT | No | Negative |
|  | PT42 | F | 44 | Kidney | Alport syndrome | Glomerulopathies | No | Negative |
|  | PT43 | F | 76 | Kidney | Renal failure | CKD | Yes | Negative |
|  | PT44 | M | 56 | Kidney | Hyperuricemia | Hyperuricaemia | No | Negative |
|  | PT45 | M | 23 | Kidney | Focal segmental glomerulosclerosis | Glomerulopathies | Yes | Negative |
|  | PT46 | M | 66 | Kidney | Renal failure | Other | No | Negative |
|  | PT47 | F | 11 | Kidney | Glomerulonephritis | Glomerulopathies | Yes | Negative |
|  | PT48 | M | 81 | Kidney | HUS | HUS | No | Negative |
|  | PT49 | F | 48 | Kidney | Gitelman syndrome | Tubulopathies | No | Negative |
|  | PT50 | F | 45 | Kidney | Alport syndrome | Glomerulopathies | Yes | Negative |
|  | PT51 | M | 10 | Kidney | Alport syndrome | Glomerulopathies | Yes | Negative |
|  | PT52 | M | 8 | Kidney | Nephronophthisis | Ciliopathies | No | Negative |
|  | PT53 | M | 64 | Kidney | Hyperuricemia | Hyperuricaemia | Yes | Negative |
|  | PT54 | F | 53 | Kidney | Tubulopathy | Tubulopathies | Yes | Negative |
|  | PT55 | M | 38 | Kidney | Nephrolithiasis | Nephrolithiasis | Yes | Negative |
|  | PT56 | F | 67 | Kidney | Focal segmental glomerulosclerosis | Glomerulopathies | Yes | Negative |
|  | PT57 | F | 45 | Kidney | Focal segmental glomerulosclerosis | Glomerulopathies | No | Negative |
|  | PT58 | F | 10 | Kidney | CAKUT | CAKUT | No | Negative |
|  | PT59 | M | 5 | Kidney | Nephrolithiasis | Nephrolithiasis | No | Negative |
|  | PT60 | F | 73 | Kidney | Renal failure | CKD | Yes | Negative |
|  | PT61 | M | 20 | Kidney | Minimal lesion glomerulonephritis | Glomerulopathies | No | Negative |
|  | PT62 | M | 45 | Kidney | Renal failure | CKD | No | Negative |
|  | PT63 | F | 12 | Kidney | CAKUT | CAKUT | No | Negative |
|  | PT64 | F | 26 | Kidney | Renal failure | CKD | Yes | Negative |
|  | PT65 | M | 7 | Kidney | Syndromic disease | CAKUT | No | Negative |
|  | PT66 | M | 21 | Kidney | Focal segmental glomerulosclerosis | Glomerulopathies | No | Negative |
|  | PT67 | M | 64 | Kidney | Hyperuricemia | Hyperuricaemia | Yes | Negative |
|  | PT68 | M | 6 | Kidney | Tubulopathy | Tubulopathies | No | Negative |
|  | PT69 | F | 9 | Kidney | Hyperoxaluria | Nephrolithiasis | No | Negative |
|  | PT70 | M | 11 | Kidney | Tubulopathy | Tubulopathies | No | Negative |
|  | PT71 | F | 17 | Kidney | Focal segmental glomerulosclerosis | Glomerulopathies | No | Negative |
|  | PT72 | M | 24 | Kidney | Ciliopathy | Ciliopathies | Yes | Negative |
|  | PT73 | F | 46 | Kidney | Nephrolithiasis | Nephrolithiasis | Yes | Negative |
|  | PT74 | M | 47 | Kidney | Tubulopathy | Tubulopathies | Yes | Negative |
|  | PT75 | F | 55 | Kidney | Focal segmental glomerulosclerosis | Glomerulopathies | No | Negative |
|  | PT76 | M | 56 | Kidney | Nephrotic syndrome | Glomerulopathies | No | Negative |
|  | PT77 | M | 2 | Kidney | CAKUT | CAKUT | No | Negative |
|  | PT78 | F | 1 | Kidney | CAKUT | CAKUT | No | Negative |
|  | PT79 | F | 5 | Kidney | CAKUT | CAKUT | No | Negative |
|  | PT80 | M | 58 | Kidney | Polycystic kidney | Ciliopathies | Yes | Negative |
|  | PT81 | M | 54 | Kidney | Cystic kidney disease | Ciliopathies | No | Negative |
|  | PT82 | M | 73 | Kidney | Polycystic kidney | Ciliopathies | No | Negative |
|  | PT83 | F | 45 | Kidney | Polycystic kidney | Ciliopathies | No | Negative |
|  | PT84 | F | 10 | Kidney | Polycystic kidney | Ciliopathies | No | Negative |
|  | PT85 | M | 11 | Kidney | Polycystic kidney | Ciliopathies | No | Negative |
|  | PT86 | M | 8 | Kidney | Polycystic kidney | Ciliopathies | Yes | Negative |
|  | PT87 | F | 1 | Kidney | Polycystic kidney | Ciliopathies | No | Negative |
|  | PT88 | M | 3 | Kidney | Polycystic kidney | Ciliopathies | Yes | Negative |
|  | PT89 | M | 8 | Kidney | Polycystic kidney | Ciliopathies | No | Negative |
|  | PT90 | M | 57 | Kidney | Cystic kidney disease | Ciliopathies | Yes | Negative |
|  | PT91 | M | 53 | Kidney | Polycystic kidney | Ciliopathies | No | Negative |
|  | PT92 | M | 55 | Kidney | Polycystic kidney | Ciliopathies | No | Negative |
|  | PT93 | F | 73 | Kidney | Cystic kidney disease | Ciliopathies | No | Negative |
|  | PT94 | M | 64 | Kidney | Cystic kidney disease | Ciliopathies | Yes | Negative |
|  | PT95 | M | 72 | Kidney | Polycystic kidney | Ciliopathies | Yes | Negative |
|  | PT96 | M | 51 | Kidney | Polycystic kidney | Ciliopathies | No | Negative |
|  | PT97 | M | 67 | Kidney | Polycystic kidney | Ciliopathies | Yes | Negative |
|  | PT98 | F | 26 | Kidney | Polycystic kidney | Ciliopathies | No | Negative |
|  | PT99 | M | 65 | Kidney | Polycystic kidney | Ciliopathies | Yes | Negative |
|  | PT100 | M | 52 | Kidney | Polycystic kidney | Ciliopathies | No | Negative |
|  | PT101 | M | 31 | Kidney | Polycystic kidney | Ciliopathies | Yes | Negative |
|  | PT102 | M | 57 | Kidney | Polycystic kidney | Ciliopathies | No | Negative |
|  | PT103 | M | 48 | Kidney | Polycystic kidney | Ciliopathies | Yes | Negative |
|  | PT104 | F | 56 | Kidney | Polycystic kidney | Ciliopathies | Yes | Negative |
|  | PT105 | M | 57 | Kidney | Polycystic kidney | Ciliopathies | Yes | Negative |
|  | PT106 | F | 50 | Kidney | Cystic kidney disease | Ciliopathies | No | Negative |
|  | PT107 | F | 40 | Kidney | Polycystic kidney | Ciliopathies | No | Negative |
|  | PT108 | F | 65 | Kidney | Cystic kidney disease | Ciliopathies | Yes | Negative |
|  | PT109 | M | 1 | Liver | Neonatal biliary atresia | Cholestasis | No | Negative |
|  | PT110 | F | 15 | Liver | Liver cirrhosis | Cirrhosis | No | Negative |
|  | PT111 | M | 0 | Liver | Cholestasis | Cholestasis | No | Negative |
|  | PT112 | F | 20 | Liver | Alagille syndrome | Cholestasis | No | Negative |
|  | PT113 | M | 17 | Liver | Hepatic ciliopathy | Ciliopathies | No | Negative |
|  | PT114 | M | 21 | Liver | Wilson disease | Other | No | Negative |

| **Table S2. In-silico panels of kidney disease-associated genes.** In bold are indicated the genes added following the revision and update of the in-silico panel lists used for the re-analysis of NGS data and absent at the time of first analysis. | |
| --- | --- |
| **GENE PANEL ID** | **GENE LIST** |
| **CILIOPATHIES (96 genes)** | ***AHI1****; ALMS1; ANKS6; ARL13B; ARL3; ARL6; B9D1; B9D2; BBIP1; BBS1; BBS10; BBS12; BBS2; BBS4; BBS5; BBS7; BBS9; BICC1; C2CD3; C5orf42 (CPLANE1); C8orf37 (CFAP418); CC2D2A; CCDC28B; CEP120; CEP164; CEP290; CEP41; CEP83; CRB2; CSPP1; DCDC2; DDX59; DHCR7; DYNC2H1; DYNC2I2; EVC; EVC2; FAN1; GLI3; GLIS2; GLIS3;* ***HNF1B****; HSD17B4; HYLS1;* ***ICK*** *(CILK1); IFT122; IFT140; IFT172; IFT27; IFT43; IFT80; INTU; INVS; IQCB1; KIF14; KIF7; LZTFL1; MKKS; MKS1; MUC1; NEK1; NEK8; NPHP1; NPHP3; NPHP4; OFD1; PDE6D;* ***PIBF1****; PKD1; PKD2; PKHD1; PMM2; REN; RPGRIP1L; SBDS; SDCCAG8;* ***SEC63****; TCTN1; TCTN2; TCTN3; TMEM138; TMEM216; TMEM231; TMEM237; TMEM67; TRAF3IP1; TRIM32; TTC21B; TTC8; UMOD; WDPCP; WDR19; WDR35; WDR60 (DYNC2I1); XPNPEP3; ZNF423* |
| **RENAL CYSTIC DISEASE (86 genes)** | ***AHI1****; ALG8; ALG9; ALMS1; ANKS6; ARL13B; ARL6; B9D1; B9D2; BBS1; BBS10; BBS12; BBS2; BBS4; BBS5; BBS7; BBS9; C5orf42 (CPLANE1); CC2D2A; CCND1; CDC73; CEP164; CEP290; CEP41; CEP83; COL4A1; CRB2; CSPP1; DDX59; DHCR7; DYNC2H1; GLIS2;* ***HNF1B****; HYLS1;* ***ICK*** *(CILK1); IFT122; IFT140; IFT172; IFT27; IFT43; INVS; IQCB1; KIF14; KIF7; LRP5; LZTFL1; MKKS; MKS1; NEK1; NEK8; NOTCH2; NPHP1; NPHP3; NPHP4; OFD1; PIGT; PKD1; PKD2; PKHD1; PMM2; PRKCSH; RAD51C; RPGRIP1L; SDCCAG8;* ***SEC63****; TCTN1; TCTN2; TCTN3; TMEM138; TMEM216; TMEM231; TMEM237; TMEM67; TRAF3IP1; TSC1; TSC2; TTC21B; TTC8; UMOD; VHL; WDPCP; WDR19; WDR35; WDR60 (DYNC2I1); XPNPEP3; ZNF423* |
| **POLYCYSTIC DISEASE (3 genes)** | *PKD1; PKD2; PKHD1* |
| **GLOMERULOPATHIES (75 genes)** | *ACTN4; ADAMTS13; ALG1; ANLN; APOA1; APOE; APOL1; ARHGAP24; ARHGDIA; B2M; C1QC; C3;* ***C4A****;* ***CD151****; CD2AP; CD46;* ***CD59****; CFB; CFH; CFHR1; CFHR3; CFHR5; CFI; COL4A3; COL4A4; COL4A5; COQ2; COQ6; COQ8B; CRB2; CUBN; DGKE; EMP2;* ***ERCC6****;* ***ERCC8****; FAT1; FGA; FN1; FOXC2; GLA; GSN; INF2; ITGA3; ITGB4; KANK2;* ***KANK4****; LAMB2; LCAT; LMNA; LMX1B; LYZ; MAGI2;* ***MMACHC****;* ***MMADHC****; MYH9; MYO1E; NPHS1; NPHS2; NUP107;* ***NXF5****; PAX2;* ***PDSS2****; PIGA; PLCE1; PTPRO;* ***SCARB2****; SMARCAL1; SPRY2; THBD; TP53RK; TRPC6; TTR; WDR73; WT1; XPO5* |
| **CAKUT (145 genes)** | *ACE; ACTB; ACTG1;* ***ACTG2****; AFF3; AGT; AGTR1; ALMS1; AMER1; ANOS1; ATP7A;* ***BCOR****;* ***BICC1****;* ***BMP4****;* ***BNC2****;* ***BSND****;* ***CCBE1****; CCNQ; CD151;* ***CDKN1C****;* ***CDX2****; CHD1L; CHD7;* ***CHRM3****;* ***CHRNA3****;* ***CHST14****; CISD2;* ***DACT1****; DCHS1; DHCR7; DSTYK;* ***DYRK1A****; ESCO2; ETFA; ETFB; ETFDH; EYA1; FANCA; FANCB;* ***FANCC****; FANCD2; FANCE; FANCI; FANCL; FAT4; FGF10; FGF20; FGFR2; FLNA; FOXC1; FOXP1; FRAS1; FREM1; FREM2; GATA3; GDF6; GLI3; GPC3; GRIP1; HNF1B; HOXA13; HPSE2;* ***HSPA9****; HYLS1; ITGA3; ITGA8; JAG1; JAM3; KAT6B; KCTD1;* ***KDM6A; KIF14****; KMT2D; KRAS;* ***KYNU; LIFR****; LMNA; LRIG2; LRP4; MUC1;* ***MYOCD; NEK8; NFIA****; NIPBL; NOTCH2;* ***NPHP3; NPNT****; NRIP1; NSDHL;* ***OCRL****; OFD1; PAX2;* ***PBX1****; PEX1; PEX5; PIGL; PIGN; PIGO; PIGV; PMM2; PORCN; PROK2; PTPN11;* ***RAD51C****; RAI1; RECQL4; REN; RET;* ***ROBO1****; ROBO2; ROR2;* ***RPGRIP1L****; SALL1; SALL4; SEMA3E; SETBP1; SF3B4; SHH; SIX1; SIX5;* ***SLX4; SOX11****; SOX17; SOX9; SRCAP; STRA6;* ***TBC1D1****; TBX18; TFAP2A; TMCO1; TNXB; TP63; TRAP1;* ***UBR1; UMOD****; UPK3A;* ***WDR60****; WFS1; WNT3; WNT4; WNT5A;* ***XRCC2****; ZIC3; ZMPSTE24;* ***ZMYM2*** |
| **TUBULOPATHIES (62 genes)** | ***ABCG2; ALDOB; AP2S1;*** *AQP2; ATP1A1; ATP6V0A4; ATP6V1B1; AVPR2; BSND; CA2; CASR; CLCN5; CLCNKA; CLCNKB; CLDN16; CLDN19; CNNM2; CTNS; CUL3;* ***CYP24A1****; DMP1; EGF; EHHADH; ENPP1;* ***FAH****; FGF23; FXYD2;* ***GATM; GNA11****; HNF1B; HNF4A; KCNA1; KCNJ1; KCNJ10; KLHL3; MUC1;* ***NR3C2****; OCRL; PHEX; REN;* ***SARS2****; SCNN1A; SCNN1B; SCNN1G; SLC12A1; SLC12A3; SLC1A1; SLC22A12; SLC2A2; SLC34A1;* ***SLC34A3; SLC4A1; SLC4A4****; SLC5A2; SLC7A7;* ***SLC9A3R1****; TRPM6; UMOD;* ***VIPAS39****;* ***VPS33B****; WNK1;* ***WNK4*** |
| **NEPHROLITHIASIS (47 genes)** | *ADCY10; AGXT; ALPL; AP2S1; APRT; ATP6V0A4; ATP6V1B1; ATP7B; BSCL2; CA2; CASR; CLCN5; CLCNKB; CLDN16; CLDN19; CTNS; CYP24A1;* ***FAH****; FAM20A;* ***G6PC*** *(G6PC1); GNA11; GRHPR; HNF4A; HOGA1; HPRT1; KCNJ1; MOCOS; OCRL; PIGT; SLC12A1; SLC22A12; SLC26A1; SLC2A2; SLC2A9; SLC34A1; SLC34A3; SLC36A2; SLC3A1; SLC4A1; SLC6A19; SLC6A20; SLC7A9; SLC9A3R1;* ***VIPAS39; VPS33B****; XDH;* ***ZNF365*** |
| **HYPERURICEMIA (21 genes)** | ***ABCG2****; ACADS; ALDOB; APRT; G6PC (G6PC1); GCKR; HNF1B; HPRT1; MUC1; PFKM; PRPS1; PYGM; REN;* ***SARS2; SLC17A1; SLC17A3****; SLC22A11; SLC22A12; SLC2A9;* ***SLC37A4****; UMOD* |
| **HEMOLYTIC-UREMIC SYNDROME (30 genes)** | *C1QA; C1QB; C1QC; C1S;* ***C2****; C3;* ***C4A; C4B****; C4BPA;* ***C5; C6; C7; C8A; C8B; C9; CD46****;* ***CD59****; CFB; CFH; CFHR1; CFHR3;* ***CFHR4; CFHR5****; CFI; DGKE; MMACHC; MMADHC; MTHFD1; PIGA; THBD* |
| **KIDNEY FULL LIST (523 genes)** | ***ABCD4; ABCG2; ACADS****; ACE; ACTB;* ***ACTG1; ACTG2****; ACTN4;* ***ADA; ADAMTS13****;* ***ADCY10****; AGT; AGTR1; AGXT; AHI1; ALDOB;* ***ALG1; ALG8; ALG9****; ALMS1;* ***ALPL****;* ***AMER1****; ANKS6;* ***ANLN****; ANOS1; AP2S1; APOA1; APOE; APOL1; APRT; AQP2;* ***ARHGAP24****; ARHGDIA; ARL13B;* ***ARL3****; ARL6;* ***ATP1A1****; ATP6V0A4; ATP6V1B1;* ***ATP7A; ATP7B; AUH; AVP****; AVPR2; B2M;* ***B3GLCT; B4GAT1****; B9D1; B9D2;* ***BBIP1****; BBS1; BBS10; BBS12; BBS2; BBS4; BBS5; BBS7;* ***BCOR; BCS1L****; BICC1;* ***BMP4****; BMPER; BNC2; BRAF; BSCL2; BSND; BTK;* ***BUB1B; C1QA; C1QB; C1QC; C1S; C2;*** *C2CD3; C3;* ***C4A; C4B; C4BPA; C5;*** *C5orf42 (CPLANE1);* ***C6; C7; C8A; C8B; C8orf37*** *(CFAP418);* ***C9****; CA2;* ***CASP10****; CASR; CC2D2A;* ***CCBE1; CCDC28B; CCND1; CD151****; CD2AP;* ***CD46****;* ***CD59; CD81; CD96; CDC5L; CDC73; CDKN1C; CEP120****; CEP164; CEP290; CEP41; CEP83; CFB; CFH; CFHR1; CFHR3;* ***CFHR4; CFHR5****; CFI;* ***CHD1L; CHD7; CHRM3****; CHRNA3;* ***CHST14; CISD2****; CLCN5;* ***CLCNKA****; CLCNKB; CLDN16; CLDN19; CNNM2;* ***COG1; COL18A1****; COL4A1; COL4A3; COL4A4; COL4A5; COL4A6;* ***COLEC11****; COQ2; COQ6; COQ8B;* ***COQ9; COX14; COX7B; CPT2****; CRB2; CSPP1; CTNS;* ***CUBN****; CUL3; CYP24A1;* ***CYP27B1; CYP2R1; CYP3A4; DCDC2; DCHS1; DDX59****; DGKE; DHCR7; DIS3L2;* ***DKC1; DLL4****; DMP1;* ***DNA2; DNASE1L3; DPH1****; DSTYK; DYNC2H1;* ***EBP****; EGF;* ***EHHADH; EIF2AK3; ELP1****; EMP2; ENPP1;* ***ERBB3; ERCC4; ERCC6; ERCC8; ESCO2;*** ***ETFA; ETFB; ETFDH; EVC; EVC2;*** *EYA1;* ***FAH; FAM20A; FAM20C; FAN1****; FANCA; FANCB;* ***FANCC; FANCD2; FANCE; FANCI; FANCL; FAT1; FAT4; FBXL4****; FGA;* ***FGF10;*** ***FGF20****; FGF23; FGFR1;* ***FGFR2; FGFR3; FLNA; FN1; FOXC1; FOXC2; FOXF1****; FRAS1;* ***FREM1****; FREM2; FXYD2;* ***G6PC*** *(G6PC1);* ***GALNT3;*** *GATA3;* ***GATM; GCKR;*** *GLA;* ***GLB1****; GLI3; GLIS2;* ***GLIS3; GNA11****; GPC3; GRHPR; GRIP1;* ***GSN; H19; HBB; HES7; HGD;*** ***HNF1A****; HNF1B;* ***HNF4A****; HOGA1****; HOXA13; HOXD13; HPRT1; HPS1****; HPSE2;* ***HRAS****;* ***HSD11B2; HSD17B4; HSPA9;*** *HYLS1;* ***ICK*** *(CILK1);* ***IFNG****; IFT122; IFT140; IFT172;* ***IFT27****; IFT43; IFT80; INF2;* ***INTU****; INVS; IQCB1;* ***ITGA3; ITGA6;*** *ITGA8; ITGB4;* ***JAG1****;* ***JAM3; KANK1; KANK2; KANK4; KANSL1;*** *KAT6B; KCNA1; KCNJ1; KCNJ10; KCTD1; KIF14; KIF7;* ***KL****; KLHL3;* ***KMT2D; KRAS; KYNU;*** *LAMB2;* ***LARS1; LCAT; LDHA; LFNG****;* ***LIFR; LMNA;*** *LMX1B;* ***LPIN1****; LRIG2;* ***LRP2; LRP4; LRP5; LTBP4;*** *LYZ; LZTFL1;* ***MAFB****;* ***MAGI2; MASP1; MBTPS2; MCM5; MEFV; MESP2****; MKKS; MKS1;* ***MLH1; MMACHC****;* ***MMADHC****; MNX1; MOCOS; MTHFD1; MUC1;* ***MUT*** *(MMUT); MVK;* ***MYCN****; MYH9; MYO1E;* ***MYOCD****; NAA10;* ***NBN; NDUFAF3****; NEK1; NEK8;* ***NFIA; NIPBL****; NLRP3; NOTCH2; NPHP1; NPHP3; NPHP4; NPHS1; NPHS2; NR3C2;* ***NRIP1; NSDHL; NUP107****;* ***NXF5****; OCRL; OFD1;* ***OPLAH****; PAX2;* ***PBX1; PC;*** *PDE6D;* ***PDSS2****; PEX1;* ***PEX10; PEX19****;* ***PEX2; PEX5; PEX6****; PFKM; PGK1; PHEX; PHGDH; PIBF1;* ***PIGA; PIGL; PIGN; PIGO****; PIGT; PIGV; PKD1; PKD2; PKHD1; PLCE1; PMM2;* ***POR; PORCN; PPP3CA; PRKAG2;*** ***PRKCSH; PRODH; PROK2; PRPS1****; PTHB1 (BBS9);* ***PTPN11****; PTPRO;* ***PUF60****;* ***PYGM****; RAB23; RAD21; RAD51C;* ***RAI1; RBM10****; RBM8A; RECQL4; REN;* ***RERE****; RET;* ***RMND1****; ROBO2;* ***ROR2****; RPGRIP1L;* ***RPL26; RPS19; RPS24; RRM2B****; SALL1;* ***SALL4; SARS2;*** *SBDS;* ***SC5D; SCARB2****; SCNN1A; SCNN1B; SCNN1G; SDCCAG8; SDHB;* ***SDHD; SEC63****;* ***SEMA3E; SETBP1; SF3B4; SHH; SI****; SIX1;* ***SIX2****; SIX5; SLC12A1; SLC12A3;* ***SLC17A1****;* ***SLC17A3; SLC17A5****; SLC1A1;* ***SLC22A11;*** *SLC22A12;* ***SLC26A1; SLC26A3****; SLC2A2;* ***SLC2A9****; SLC34A1; SLC34A3; SLC36A2;* ***SLC37A4****; SLC3A1; SLC4A1; SLC4A4; SLC5A2;* ***SLC6A19; SLC6A20****; SLC7A7; SLC7A9; SLC9A3R1;* ***SLX4****; SMARCAL1;* ***SMC1A; SNRPB****;* ***SOX11****; SOX17;* ***SOX18; SOX9; SPECC1L; SPRY2; SRCAP; STAR; STRA6; TBX15; TBX18****; TCTN1; TCTN2; TCTN3;* ***TFAP2A;*** *THBD;* ***THOC6; TMCO1;*** *TMEM138; TMEM216; TMEM231; TMEM237; TMEM67; TNXB;* ***TP53RK; TP63****; TRAF3IP1;* ***TRAP1****; TRIM32; TRPC6; TRPM6; TSC1; TSC2; TTC21B; TTC37; TTC8;* ***TTR; TXNL4A****; UBE3B;* ***UBR1****; UMOD; UMPS;* ***UPB1****; UPK3A;* ***USP9X****; VANGL1; VDR; VHL; VIPAS39; VPS33B;* ***WAS; WASHC5;*** *WDPCP; WDR19; WDR34 (DYNC2I2); WDR35; WDR60 (DYNC2I1); WDR73; WFS1; WNK1; WNK4;* ***WNT3; WNT4****; WNT5A; WT1; XDH;* ***XPNPEP3; XPO5; XRCC2; XRCC4; XYLT1; YWHAE; ZIC3; ZMPSTE24; ZNF365****; ZNF423* |

| **Table S3. In-silico panels of liver disease-associated genes.** In bold are indicated the genes added following the revision and update of the in-silico panel lists used for the re-analysis of NGS data and absent at the time of first analysis. | |
| --- | --- |
| **GENE PANEL ID** | **GENE LIST** |
| **CHOLESTASIS AND BILE ACID DISORDERS (26 genes)** | *ABCB11; ABCB4;* ***ABCC12; ABCC3; ABCD3;*** *AKR1D1;* ***AMACR; AP1S1****; ATP7A; ATP7B;* ***ATP8B1;*** *BAAT;* ***CYP27A1; CYP7B1;*** *EPHX1;* ***HSD3B7****; JAG1; NOTCH2; NR1H4; SLC10A1; SLC30A10; SLC33A1; SLCO1A2; TJP2; VIPAS39; VPS33B* |
| **HEPATIC CILIOPATHIES (130 genes)** | ***ACVR2B; ADGRV1****; AHI1;* ***AIPL1; ALMS1; ANKS6****; ARL13B; ARL6; ARMC4 (ODAD2);* ***ATXN10; B9D1****; B9D2; BBIP1; BBS1; BBS10; BBS12; BBS2; BBS4; BBS5; BBS7; BBS9; C21orf2 (CFAP410); C21orf59 (CFAP298); C2CD3; C2orf71 (PCARE); C5orf42 (CPLANE1); C8orf37 (CFAP418); CC2D2A;* ***CCDC103; CCDC114*** *(ODAD1); CCDC151 (ODAD3); CCDC28B; CCDC39; CCDC40; CCDC65; CCNO; CDH23; CEP120; CEP164; CEP290; CEP41; CEP83;* ***CFAP53****; CFC1; CFTR; CLRN1; CRB1; CRB2; CRELD1; CRX; CSPP1; DCDC2; DDX59; DHCR7; DNAAF1;* ***DNAAF2; DNAAF3; DNAAF4; DNAAF5; DNAH1; DNAH11; DNAH5; DNAI1; DNAI2; DNAL1; DRC1****; DYNC2H1; EVC; EVC2;* ***EXOC8; FOXH1; GDF1; GLI3; GLIS2; GUCY2D****; HNF1B; HYDIN; HYLS1; ICK (CILK1); IFT122; IFT140; IFT172; IFT27; IFT43; IFT80;* ***IMPDH1; INVS****; IQCB1;* ***KCNJ13; KIF14****; KIF7;* ***LBR; LCA5****; LEFTY2;* ***LRAT; LRRC6*** *(DNAAF11); LZTFL1; MKKS; MKS1;* ***MUC1****;* ***MYO7A****; NEK1; NEK8;* ***NKX2-5; NME8; NODAL****; NPHP1; NPHP3; NPHP4;* ***OCRL****; OFD1;* ***PCDH15; PDE6D; PIBF1****; PKD1; PKD2; PKHD1; PMM2;* ***POC1A****; POC1B;* ***PRKCSH; RD3; RDH12; RPE65; RPGR****; RPGRIP1; RPGRIP1L;* ***RSPH1; RSPH4A; RSPH9****; SBDS;* ***SCLT1; SCNN1A; SCNN1B****; SCNN1G; SDCCAG8;* ***SEC63; SPAG1; SPATA7****; SUFU; TCTN1; TCTN2; TCTN3; TMEM138; TMEM216; TMEM231; TMEM237; TMEM67;* ***TOPORS; TRAF3IP1; TRIM32; TSC1; TSC2; TTBK2****; TTC21B; TTC8;* ***TULP1****;* ***UMOD; USH1C; USH1G; USH2A; VHL****; VPS13B; WDPCP; WDR19; WDR34 (DYNC2I2); WDR35; WDR60 (DYNC2I1);* ***WHRN; XPNPEP3; ZIC3; ZMYND10****; ZNF423; ZSWIM6* |
| **LIVER FULL LIST (273 genes)** | *AAAS, ABCA1, ABCB11, ABCB4, ABCC2, ABCC3, ABCD3, ABCG5, ABCG8, ABHD5, ADK, AGL, AGPS, AGXT, AKR1D1, ALAD, ALDOA, ALDOB, ALG1, ALG11, ALG12, ALG13, ALG2, ALG3, ALG6, ALG8, ALG9, ALMS1, AMACR, ANKS6, AP1S1, APOA1, APOA5, APOB, APOC2, APOC3, APOE, ARG1, ARL13B, ARL3, ASL, ASS1, ATP7A, ATP7B, ATP8B1, B2M, B4GALT1, B9D2, BAAT, BCKDHA, BCKDHB, CC2D2A, CCBE1, CEP164, CEP290, CEP41, CEP83, CFC1, CFTR, CLDN1, COG1, COG2, COG4, COG5, COG6, COG7, COG8, CPS1, CPT2, CSPP1, CTRC, CYP27A1, CYP7A1, CYP7B1, DBT, DCDC2, DDOST, DGUOK, DHCR7, DHDDS, DOLK, DPAGT1, DPM1, DPM2, EHHADH, EIF2AK3, EPHX1, FAH, FAT4, FBXL4, FGA, G6PC, GAA, GALT, GBA, GBE1, GLIS3, GNAS, GNPAT, GPD1, GPIHBP1, GYS2, HADHA, HAMP, HFE, HFE2, HMBS, HNF1A, HNF1B, HPD, HSD17B4, HSD3B7, IFT43, INVS, JAG1, KIF7, LARS, LDLR, LDLRAP1, LIPA, LPL, LRP5, LRPPRC, LYZ, MARS, MGAT2, MGME1, MKS1, MOGS, MPDU1, MPI, MPV17, MUT, MVK, MYO5B, NAGS, NBAS, NEUROG3, NOTCH2, NPC1, NPC2, NPHP3, NR1H4, OTC, PC, PCCA, PCCB, PCSK9, PEX1, PEX10, PEX11B, PEX12, PEX13, PEX14, PEX16, PEX19, PEX2, PEX26, PEX3, PEX5, PEX6, PEX7, PFKM, PGM1, PHKA1, PHKA2, PHKB, PHKG2, PIBF1, PKHD1, PLIN1, PMM2, PNPLA2, PNPLA3, POLG, PPOX, PRKCSH, PRSS1, PYGL, PYGM, RFT1, RHAG, RPGRIP1L, RRM2B, SEC63, SERAC1, SERPINA1, SKIV2L, SLC10A1, SLC10A2, SLC25A13, SLC25A15, SLC25A19, SLC27A5, SLC30A10, SLC33A1, SLC35A1, SLC35A2, SLC35C1, SLC37A4, SLCO1A2, SLCO1B1, SLCO1B3, SLCO2B1, SMPD1, SPINK1, SRD5A3, SSR4, STT3A, STT3B, SUCLA2, SUCLG1, TALDO1, TAT, TCTN2, TFR2, TJP2, TMEM165, TMEM216, TMEM67, TRMU, TTC37, TTR, TWNK, UGT1A1, UROS, UTP4, VIL1, VIPAS39, VPS33B,* ***ABCC12, ACAD9, ACOX1, ANGPTL3, APOA2, BCS1L, BRAF, CBL, CETP, EPHX2, FBP1, GNE, HFE2, HRAS, KRAS, LCAT, LIPC, LZTR1, MAP2K1, MAP2K2, MTTP, MUT, NF1, NGLY1, NRAS, PIGA, PIGM, PKD1, PKD2, POLG2, PTPN11, RAF1, RASA2, RBCK1, RIT1, RRAS2, SAR1B, SCO1, SHOC2, SOS1, SOS2, SPRED1, TFAM, TRAPPC11, TRIM37*** |

| **Table S4. List of primers used for Sanger validation.** | | |
| --- | --- | --- |
| **GENES** | PRIMER_FORWARD | PRIMER_REVERSE |
| ***ALG8* (c.1507A>G)** | AGGAAATGTTTGCTAAACCAGGA | GTCTTGCCAATAGCAGAGTCA |
| ***ALG8* (c.154A>G)** | TCTGACCTCCAGATAAATGAGGTTT | AACTTACCTCATAATACCACTGTGA |
| ***ZNF423* (c.2849A>C)** | CCTACACCATGGAGGTGCTG | ATCTTGCAGATGCGACAGGT |
| ***CDKN1C* (c.73G>A)** | GACGCAGAAGAGTCCACCAC | CATGTCCTGCTGGAAGTCGT |
| ***ROBO2* (c.2297A>G)** | AGTAGCATGAACGCACACCA | GCATTTGTTTCATTCTCTGTCACAC |
| ***PKD2* (c.1185G>C)** | TGCCTCAAGTGTTCCACTGAT | AGCTAACTGCAGGCAAAGGT |
| ***PBX1* (c.712C>T)** | TAGCGTTGGTTTTGGCATCC | TGCTTACCTGGGAGACTGTG |
| ***NOTCH2* (c.6028-5T>A)** | ACATTGGTAGAGGCACAGCC | TGCTTCAGGCTGAGGAAAGA |
| ***COL4A3* (c.-10C>T)** | CAACCAAAAAGCCTGGGTGG | CGTGGAGGAGGGATGGAAGT |
| ***KMT2D* (c.4031T>C)** | TTCCTGGCTTTTGGCCTTCA | TCCCCAAAGTAGGTCCAGTT |
